# Foraging ecology of Eurasian lynx populations in southwest Asia: Conservation implications for a diet specialist

**DOI:** 10.1002/ece3.4439

**Published:** 2018-08-29

**Authors:** Deniz Mengüllüoğlu, Hüseyin Ambarlı, Anne Berger, Heribert Hofer

**Affiliations:** ^1^ Department of Evolutionary Ecology Leibniz Institute for Zoo and Wildlife Research (IZW) Berlin Germany; ^2^ Department of Wildlife Ecology and Management Faculty of Forestry Düzce University Düzce Turkey; ^3^ Department of Ecological Dynamics Leibniz Institute for Zoo and Wildlife Research Berlin Germany; ^4^ Department of Veterinary Medicine Freie Universität Berlin Berlin Germany; ^5^ Department of Biology, Chemistry, Pharmacy Freie Universität Berlin Berlin Germany

**Keywords:** brown hare, cannibalism, feeding behavior, functional response, prey preferences, Turkey

## Abstract

Intraspecific variation in key traits of widespread species can be hard to predict, if populations have been very little studied in most of the distribution range. Asian populations of the Eurasian lynx (*Lynx lynx*), one of the most widespread felids worldwide, are such a case in point. We investigated the diet of Eurasian lynx from feces collected Mediterranean, mixed forest‐steppe, and subalpine ecosystems of Turkey. We studied prey preferences and functional responses using prey densities obtained from Random Encounter Modelling. Our analysis revealed that the main prey was brown hare (*Lepus europaeus*) in all three areas (78%–99% of biomass consumed) and lynx showed a strong preference for brown hare (Chesson's selectivity index, *α* = 0.90–0.99). Cannibalism contributed at least 5% in two study areas. The type II functional response of lynx populations in Turkey was similar to the Canada lynx (*Lynx canadensis*) and daily food intake in grams per lynx matched that of Canada lynx and Iberian lynx (*Lynx pardinus*), both lagomorph specialists, rather than those of Eurasian lynx from Europe. Therefore, lynx in Turkey may be better described as a lagomorph specialist even though it coexists with ungulate prey. We suggest that ungulate‐based foraging ecology of Eurasian lynx in Europe may be a recent adjustment to the availability of high densities of ungulates and cannot be representative for other regions like Turkey. The status of lagomorphs should become an essential component of conservation activities targeted at Eurasian lynx or when using this species as a flagship species for landscape preservation.

## 1 INTRODUCTION

Assigning a certain trait to a particular population and generalizing it toward the species can cause oversimplification errors, especially in case of widespread species which experience substantial variation in environmental conditions and habitats (Putman & Flueck, 2011). Such generalizations may miss relevant variability in behavior, physiology and ecology between populations, particularly in taxa such as carnivores that show considerable interspecific and intraspecific variation (Lott, 1991; Moehlman & Hofer, 1997). These generalizations may become of practical relevance if conservation actions are built upon expectations derived from populations studied elsewhere, with the potential to fail if the biology of the local population is different, for instance because it is adapted to local and historical environmental conditions.

The Eurasian lynx (*Lynx lynx* Linnaeus, 1758) is a Palearctic species and one of several felids that have very wide distributions. Although European populations have suffered a tremendous decline, the species still covers a vast range from central Europe to central, north, and far eastern Asia. Most studies on the ecology and behavior of lynx have been conducted on populations in central and eastern Europe (e.g., Jedrzejewski, Schmidt, Miłkowski, Jędrzejewska, & Okarma, 1993; Jobin, Molinari, & Breitenmoser, 2000; Odden, Linnell, & Andersen, 2006; Okarma, Jędrzejewski, Schmidt, Kowalczyk, & Jędrzejewska, 1997; Sunde, Kvam, Bolstad, & Bronndal, 2000), with very few exceptions (Sedalischev, Odnokurtsev, & Ohlopkov, 2014; Weidong, 2010), and concluded that Eurasian lynx is a specialist predator of medium‐sized and large‐sized ungulates and hunts smaller mammals when ungulates are not available (Breitenmoser et al., 2000; Jedrzejewski et al., 1993; Odden et al., 2006). If this general hypothesis is correct, then other lynx populations, for instance in Asia, should show the same foraging ecology and feeding preferences as the central European populations. From a comparative perspective, it is noteworthy that other lynx species rarely hunt medium‐sized or large‐sized ungulates and prefer to hunt lagomorphs instead. For instance, although it is known to kill other prey species (Bergerud, 1983), the Canada lynx (*Lynx canadensis*) is considered to have specialized on hunting snowshoe hare (*Lepus americanus*), the only available medium‐sized mammal in North America present during the evolution of this lynx species (Werdelin, 1981). Similarly, the Iberian lynx (*Lynx pardinus*) is a specialist hunter of rabbits (*Oryctolagus cunniculus*). Since Eurasian lynx first evolved in Asia (Werdelin, 1981), an alternative hypothesis suggests that we should expect Eurasian lynx in Asia to be a lagomorph specialist like Canada and Iberian lynx and differ in its foraging ecology from Eurasian lynx populations in central and eastern Europe where the lynx diet is based on ungulates.

In this study, we tested both hypotheses by studying the foraging ecology and diet of Eurasian lynx (*Lynx lynx*) populations in three geographic regions of Anatolia, the Asian part of Turkey. We also compared the foraging ecology of lynx in Turkey, other Eurasian lynx populations, Canada, and Iberian lynx. Our three study areas represent major ecosystems in much of southern Europe and southwestern Asia: a Mediterranean ecosystem in the south, a mixed forest‐steppe ecosystem in the central part, and a subalpine ecosystem (Lesser Caucasus) in northeastern Anatolia. We collected fecal samples of lynx in those ecosystems where lynx is in sympatry with at least two ungulate species known to be preyed by Eurasian lynx elsewhere. Additionally, we used camera trap data to estimate prey densities and biomass to quantify prey preferences of lynx subpopulations in Turkey and compare them to European conspecifics and other lynx species. We discuss implications of these results for the design of appropriate conservation initiatives for lynx in Turkey.

## 2 MATERIAL AND METHODS

### 2.1 Study areas

#### 2.1.1 South: Mediterranean ecosystem (Antalya)

**Figure 1:**
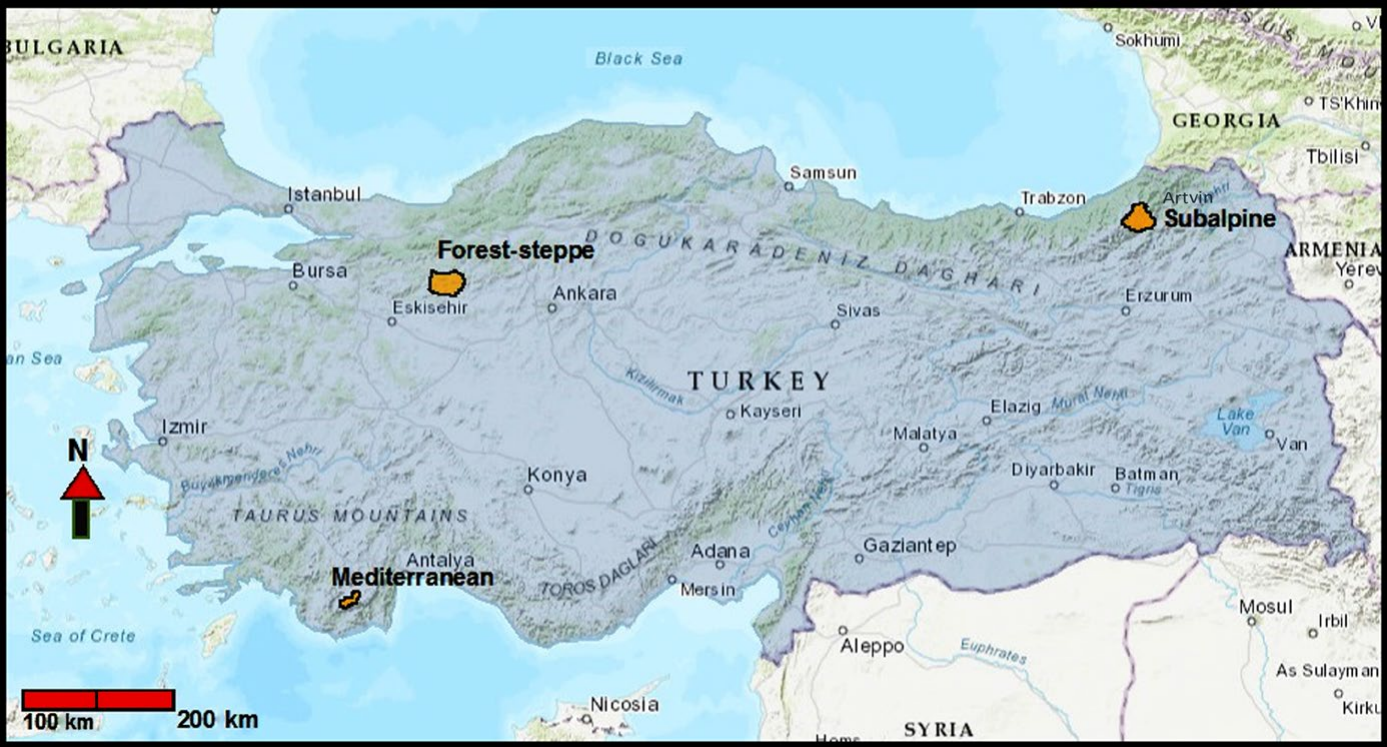
Locations of the three study areas (colored in orange) in Turkey

Fecal samples from the Mediterranean ecosystem were obtained from Antalya Çığlıkara Nature Reserve and Sedir Research Forest in Antalya (Figure 1). The study area was mostly covered by evergreen Lebanon cedar trees (*Cedrus libani*) and otherwise sparse vegetation. The study area covered 180 km^2^ at elevations between 1,290 and 3,000 m. It was close to settlements but also surrounded by high mountains and had limited access of people. Human activities were not allowed in the park area; there is no free road access. The area is known to have a high density of lynx (Avgan, Zimmermann, Güntert, Arıkan, & Breitenmoser, 2014). Potential prey species in this study area were wild goat (*Capra aegagrus*), wild boar (*Sus scrofa*), and brown hare (*Lepus europaeus*). Gray wolf (*Canis lupus*) and red fox (*Vulpes vulpes*) were two intraguild carnivores in sympatry with lynx in this area (Avgan et al., 2014).

#### 2.1.2 Central‐west: Forest‐Steppe mosaic ecosystem (Ankara)

Here, feces of lynx were collected in an area of 400 km^2^ in the Nallıhan Mountains (Figure 1). The elevation varied between 500 and 1,550 m, and the study area was located in the transition zone between the dry western Black Sea (xero‐euxine) and central Anatolian (Iran‐Turan) floristic zones. This region is also influenced by the Mediterranean floristic zone (western Aegean), through the catchment area of the Sakarya River (Aksoy, 2009). Vegetation composition and structure depended on altitude and historical human use. The lower areas (500–1,000 m) were covered by Turkish pine (*Pinus brutia*). Above this belt, temperate coniferous forest reached up to 1500 m and was composed of black pine (*Pinus nigra*), junipers (*Juniperus excelsa* and *J. oxycedrus*) with an understory of oak‐dominated scrub (*Quercus pubescens*,* Pyrus elaeagnifolia*,* Crataegus* spp., Aksoy, 2009). The human population in this area is at a low density and restricted to several villages in the surrounding lowland and valleys. The potential prey species for lynx are red deer (*Cervus elaphus*), wild boar, and brown hare. The area is home to several other large carnivores, including brown bear (*Ursus arctos)*, gray wolf, golden jackal (*Canis aureus*), red fox, and jungle cat (*Felis chaus*) (Mengüllüoğlu, 2010).

#### 2.1.3 Northeast: subalpine ecosystem (Artvin, Lesser Caucasus)

Fecal samples were collected in the Kaçkar Mountains of Artvin Province, in north eastern Turkey, in an area of 400 km^2^ (Figure 1). Our survey area covered elevation zones between 700 and 2,500 m. The vegetation changes from oak woodlands at 700–1,600 m to alpine meadows above 2,200 m with mixed dense forest, dominated by fir (*Abies nordmanniana)* and spruce (*Picea orientalis)* on northern aspects, and Scots pine (*Pinus sylvestris*) woodland on southern aspects (Ambarlı & Bilgin, 2013). Deciduous shrubland occurred mostly on the southern aspect of the mountains at drier lower elevations, whereas mixed forests were present in more humid parts. Wild goat, chamois (*Rupicapra rupicapra*), wild boar, and brown hare are potential prey of lynx in this area. Brown bear, gray wolf, golden jackal, and red fox are other carnivore species in sympatry with lynx at this area (Ambarlı & Bilgin, 2013).

### 2.2 Feces collection and diet analysis

Relatively fresh samples (*n* = 27) were opportunistically collected in the southern study area while walking on dirt roads between December 2013 and April 2014. We identified them to be of lynx origin by size, shape, and distinct odor. The only feces which could be misidentified in this area were those of red foxes (*n* = 2). We excluded two feces because they failed the lynx scat identification criteria (Kaczensky et al., 2009), namely, their segmentation and shape patterns, and had smaller diameters (1.4 and 1.5 cm).

Feces (*n* = 101) from the central‐western study area were collected by walking on active wildlife trails, dirt roads, and ridgelines in the Nallıhan Mountains between November 2013 and March 2015. In order to reduce the chance of falsely designating feces from other wild carnivores and dogs as lynx feces, a Labrador breed dog was trained to find and identify lynx feces (Smith, Ralls, Davenport, Adams, & Maldonado, 2001) in this study area, as this area is more frequently visited by domestic hunting dogs than the other two study areas.

The samples (*n* = 69) from the subalpine study area were collected randomly between 2010 and 2014 on 10 predetermined transects along trails below the tree line. They were chosen because of the long distance from settlements and human interference, lack of road access, and the absence of red foxes. The length of transects varied between 1.5 and 8.0 km (mean distance = 3.8 ± 0.7 km). They were checked on foot every year.

In addition to visual and olfactory identification, all samples collected from forest‐steppe study area (*n* = 101) and fresh samples from Mediterranean (*n* = 16) and subalpine study areas (*n* = 20) were genetically analyzed for microsatellites and confirmed to belong to lynx. However, old samples were not genetically analyzed; they matched in size and appearance the genetically identified 137 lynx samples. Lynx feces were oven‐dried and washed following the protocols of Wagner, Holzapfel, Kluth, Reinhardt, and Ansorge (2012). Prey remains such as hair, bones, teeth, nails, and feathers were separated and weighed. Hairs were classified according to their microstructure and identified with the help of reference book (Teerink, 1991) or by comparing them with local wildlife and livestock reference collections taken from the Berlin Natural History Museum (Supporting information, Table A1). After classification of fecal material, the frequency of occurrence (FO) of each species in the diet was noted and compared with the diets of lynx populations in Europe (Klare, Kamler, & Macdonald, 2011). For the purpose of estimating the consumed biomass per prey species, we used the lynx regression model of Wachter et al. (2012), which was applied to the results of European lynx feeding experiments conducted by Rühe, Burmester, and Ksinsik (2007). We calculated the consumed mass of each prey species per feces and then multiplied this value with the total ingested volumes. For the species that were not included in Rühe et al.'s (2007) experiment, we directly applied the model on average body weight of prey species and obtained consumed biomass per feces.

### 2.3 Prey preferences

Population densities and mean biomass of the prey species in the study areas have to be known in order to assess prey preferences. For the prey species where individuals cannot be individually distinguished in camera trap photographs, density estimation in forest habitats is difficult and generally gives biased results as the actual population numbers are underestimated (Jobin et al., 2000). We therefore used the Random Encounter Model (REM; Rowcliffe, Field, Turvey, & Carbone, 2008) to estimate the density *D* of mid‐sized and large herbivore prey as $D=\frac{y}{t}*\frac{\pi }{Vr(2+\theta )}$, where *y* is the number of independent photographic events, *t* is camera trap days (ctd), *V* is average speed of animal movement, and *r* and *θ* are the camera trap detection distance (in kilometer) and angle (in radian). Animal movement speeds were taken from the published literature with GPS fix frequencies of 15 min for red deer (Pépin, Adrados, Mann, & Janeau, 2004) and wild boar (Spitz & Janeau, 1990) and 1 hr for brown hare (Schai‐Braun, Rödel, & Hackländer, 2012). As there is no published data for movement speed of wild goat, we used movement speed of a wild goat collared in our subalpine study area with GPS fix frequency of 2 hr (Ambarlı, Hüseyin unpublished data).

In the model, we used the numbers of captures and camera trap days from the recent camera trapping studies in the southern and subalpine study areas (Ambarlı & Bilgin, 2013; Avgan et al., 2014; respectively). The former one placed camera traps for 1093 camera trap days on dirt roads, and in the latter one, camera traps were set up on trails in extremely rugged montane habitats for 620 camera trap days. Camera trapping data for the forest‐steppe mosaic ecosystem were gathered from a 684 camera trap days survey, implemented by us while doing feces surveys in the spring of 2014. In this survey, 12 camera trapping stations (two Cuddeback Attack, WI, USA, camera traps per station) were installed covering a minimum convex polygon of 148 km^2^. Traps were installed on forest trails and, where there was no access to interior forest due to steepness, we installed them on dirt roads. We set a minimum interval of 30 min to assign two pictures of the same species as independent captures. Camera trap detection distance and angles were obtained from Meek, Ballard, and Fleming (2012). The camera trap surveys conducted in the three different ecosystems were designed to photograph lynx, but they also photographed other carnivores and prey species. Soofi et al. (2017) showed that red deer density estimates by distance sampling and REM did not differ significantly from each other, although the camera traps were installed on leopard trails. Therefore, we assume that placements were random with respect to the movements of the three ungulate species and brown hare. We used delta method (Seber, 1982) to calculate 95% of confidence intervals for the estimated densities (Table 1).

**Table 1:** Body weights (¾ adult female live body weights for ungulates) and camera trap parameters used to calculate Random Encounter Model (REM) densities and prey biomass in three study areas in Turkey

Ecosystem	Herbivore prey	Body weight (kg)	Captures	Trap days	Travel speed (*v*)	Radius (*r*)	Angle (θ, in radians)	REM density (/km^2^); 95% Confidence intervals	Number of stations
Mediterranean	Brown hare	3.17^a^	343^c^	1,093^c^	0.890 ± 0.163^f^	0.011^j^	0.70^j^	36.15 ± 7.46; (26.69–55.52)	17^c^
	Wild boar	60^b^	26^c^		6.591 ± 3.157^g^			0.41 ± 8.56; (0.19–2.31)	
	Wild goat	30^b^	1^c^		1.580 ± 0.027^h^			0.07 ± 0.00; (0.06–0.07)	
Forest‐steppe	Brown hare	3.17^a^	508^d^	684^d^	0.890 ± 0.163^f^	0.011^j^	0.70^j^	88.27 ± 18.77; (64.94–134.92)	12^d^
	Red deer	75^b^	41^d^		3.988 ± 1.788^i^			1.59 ± 8.98; (0.80 ‐7.98)	
	Wild boar	60^b^	57^d^		6.591 ± 3.157^g^			1.34 ± 11.55; (0.61–7.34)	
Subalpine	Brown hare	3.17^a^	7^e^	620^e^	0.890 ± 0.163^f^	0.011^j^	0.73^j^	1.33 ± 0.28; (0.98–2.04)	8^e^
	Wild boar	60^b^	12^e^		6.591 ± 3.157^g^			0.31 ± 4.07; (0.14 ‐1.68)	
	Wild goat	30^b^	21^e^		1.580 ± 0.027^h^			2.24 ± 0.04; (2.16–2.33)	

*Notes*

^a^Demirbaş et al. (2013). ^b^Turan (1984). ^c^Avgan et al. (2014). ^d^This study. ^e^Ambarlı and Bilgin (2013). ^f^Schai‐Braun et al. (2012). ^g^Spitz and Janeau (1990). ^h^Ambarlı H. unpublished data. ^i^Pépin et al. (2004). ^j^Meek et al. (2012).

On the basis of the estimated densities, available mean prey biomass was calculated by using an average adult live body weight of 3.17 kg for brown hare (Demirbaş, Albayrak, & Yilmaz, 2013) and three‐fourth of female adult live body weights of 75 kg for red deer, 30 kg for wild goat, and 60 kg for wild boar to account for juveniles (Turan, 1984).

Chesson's selectivity index α (Chesson, 1978) was then used to assess lynx prey preferences. Chesson's α is defined as the proportion of prey species in the scat divided by the proportion of prey species *i* in the environment, *p*_*i*_, normalized in such a way that the sum of the alpha values over all *k* prey species equals one (Chesson, 1978).

### 2.4 Functional response

We assessed the functional response and daily food intake rates of three species of lynx preying on their favorite prey species using Holling's disc equation (Holling, 1965). These included our three lynx populations in Turkey preying on brown hare (Mediterranean, An1; Forest‐steppe, An2; Subalpine, An3), two European lynx populations preying on mountain hare (*Lepus timidus*) in Finland (Fin1, Fin2), seven Eurasian lynx populations in central and eastern Europe preying on roe deer (Eu1‐Eu7), and compared them with the lagomorph specialists Canada lynx, CL (preying on snowshoe hare) and Iberian lynx, IL (preying on rabbits). Prey intake (Ψ*,* prey intake per lynx per day) was calculated as $\left(\Psi \right)=\frac{\mathrm{aN}}{1+a(h_{1}+h_{2})N}$, where *a* is the area of effective search per unit time, *N* is the prey density, *h*_*1*_ is the time per attack multiplied by attacks per successful capture and *h*_*2*_ prey handling time which is the time period needed to consume and digest a killed prey item (Holling, 1965). Calculated prey intakes were multiplied with available carcass masses of prey to get food intake rates in grams per lynx per day (please see the Supporting information, Table A2 for references of all population‐specific parameters used in these calculations).

We used two different average daily moved distances (DMD) for Eurasian lynx in Turkey and other areas of Eurasian lynx range because of the differences in habitats and body sizes. DMD for lynx populations in Turkey was calculated from five radio‐collared lynx individuals and 15,421 GPS locations (24 fixes per day) to be 5.12 km/day (Mengüllüoğlu, D. unpublished data), whereas an average DMD of 7.2 km/day (Jedrzejewski, Schmidt, Okarma, & Kowalczyk, 2002) was used for the European populations. Densities for roe deer, mountain hare, snowshoe hare, and European rabbits were obtained from previous published work (Supporting information, Table A2). Success of attack was assumed to be the same for brown hare, and mountain hare as 35% (Pulliainen, Lindgren, & Tunkkari, 1995) and for roe deer as 66% (Pulliainen et al., 1995). As hares and rabbits are completely consumed by lynx, total adult mass was used for the calculation of biomass consumed. For roe deer, 70% of roe deer body mass was assumed to be ingested by lynx as indicated in the previous feeding studies (Okarma et al., 1997; Rühe et al., 2007; Sunde et al., 2000).

Time per attack on hares was assumed to be the same as the attack time of Canada lynx on snowshoe hare, that is, 33 s (Pulliainen, 1981), and 30 s for Iberian lynx on European rabbit (Supporting information, Table A2). Time per digestion for brown hare was estimated from cluster data for five lynx individuals from Anatolia to be 3 days on average (Mengüllüoğlu, D. unpublished data) and 2 days of digestion for Finnish lynx populations (Pulliainen, 1981), 2 days for Canada lynx and 1 day for Iberian lynx (Supporting information, Table A2). An average number of 6 days was used for European lynx populations consuming and digesting roe deer (Jobin et al., 2000). A sensitivity analysis (Burgman, Ferson, & Akçakaya, 1993) was carried out to assess which parameters in Holling's disc equation had a strong influence on Ψ, by applying Beck's Rule (Beck, 1983). Ψ was regarded as highly sensitive to a given input parameter if a 10% change in the value of the input parameter led to a change in Ψ which exceeded 10%, and showed low sensitivity if the change in Ψ was less than 10%.

## 3 RESULTS

### 3.1 Diet of lynx populations in Turkey

**Figure 2:**
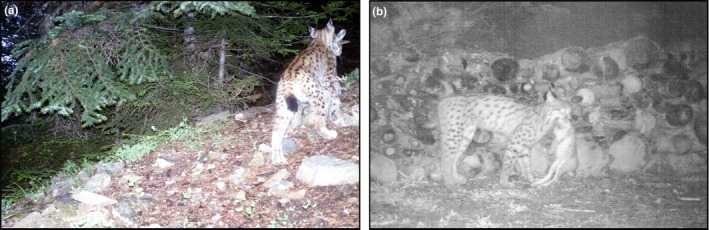
Camera trap photographs of lynx with killed brown hare in (a) subalpine study area in northeastern Turkey and (b) forest‐steppe study area in central‐west Turkey

**Figure 3:**
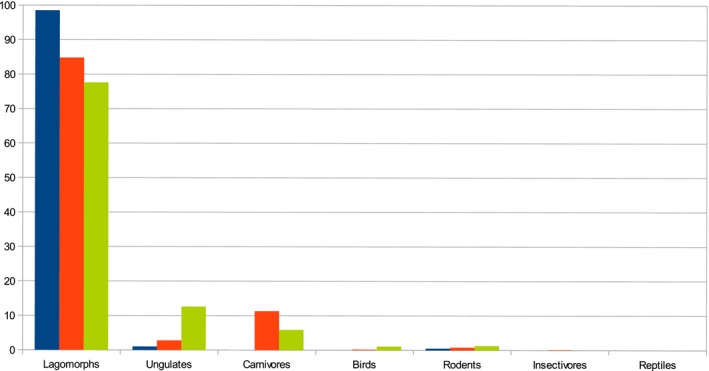
Percentages of consumed biomass in three lynx diets. Blue ‐ Mediterranean, red ‐ forest‐steppe, and green ‐ subalpine study areas

A total of 22 different prey species were identified from 256 food remains in 195 feces (Table 2). Eurasian lynx in the Mediterranean ecosystem had five prey items (Table 2). The most diverse diets belonged to Eurasian lynx in forest‐steppe and subalpine ecosystems, with 16 and 15 different prey items, respectively, including unidentified birds and rodents as two items (<5%). The number of prey items was higher in the two northern ecosystems (forest‐steppe and subalpine) as here the fecal samples contained small mammals, birds, and several carnivore species. The main prey of lynx was the brown hare in all ecosystems (Figure 2). Relative frequencies of occurrence of brown hare expressed as percentages were very high and quite similar: 100% for the Mediterranean study area, 86% for the forest‐steppe study area, and 89% for the subalpine study area (Table 2). In terms of relative total biomass consumed, brown hare constituted 99% of the diet in the Mediterranean study area, 85% in the forest‐steppe study area, and 78% in the subalpine study area (Figure 3).

**Table 2:** Diet from feces of Anatolian lynx in three different study areas in Turkey, expressed as frequency of occurrences (FO), relative frequency of occurrences (%FO), relative volume (% Vol), and relative biomass (% Bio)

Prey	kg consumed per feces	Mediterranean (*n* = 25)				Forest‐Steppe Mosaic (*n* = 101)				Subalpine (*n* = 69)			
		FO	% FO	% Vol	% Bio	FO	%FO	% Vol	% Bio	FO	% FO	% Vol	% Bio
*Capra aegagrus*	1.03									4	6.2	5.9	**8.2**
*Sus scrofa*	0.49*/0.78	1	4	1.6	1.0*	3	3	2.7	2.8	1	1.5	1.4	1.5
*Lepus europaeus*	0.77	25	100	96.9	**98.5**	86	86	81.4	**84.8**	58	89.2	74.5	**77.6**
*Lynx lynx*	0.8					10	10	8.2	**8.9**	6	9.2	5.4	**5.8**
*Canis aureus*	0.8					1	1	0.7	0.7				
*Vulpes vulpes*	0.61									1	1.5	0.1	0.1
*Martes foina*	0.2									1	1.5	0.1	0
*Sciurus anomalus*	0.2	1	4	1	0.3	2	2	0.3	0.1				
*Sciurus vulgaris*	0.2									5	7.7	1.5	0.5
*Glis glis*	0.2									1	1.5	0.8	0.2
*Dryomys nitedula*	0.2					1	1	0	0				
*Muscardinius avellanarius*	0.2					1	1	0.3	0.1	2	3.1	0.6	0.2
*Apodemus sp*.	0.2	2	8	0.3	0.1	2	2	0.4	0.1	1	1.5	0	0
*Microtus sp*.	0.2					7	7	1.7	0.5	5	7.7	0.8	0.2
*Myodes glareolus*	0.2					1	1	0.1	0				
*Crocidura sp*.	0.2					1	1	0.2	0.1				
Unidentified rodent	0.2	1	4	0.2	0.1	1	1	0	0	7	10.8	0.6	0.2
*Tetraogallus caspius*	0.46									3	4.6	2.2	0.8
Unidentified bird	0.07					3	3	2	0.2	3	4.6	2.7	0.3
*Testudo gracea*	0.14					1	1	0.2	0				
Domestic prey													
*Canis familiaris*	0.93					1	1	1	1.2				
*Felis catus*	0.37					1	1	1	0.5				
*Capra hircus*	1.03									2	3.1	3.1	4.4

*Note*

Bold percentages indicate Bio >5% ; * the correction factor for piglets.

### 3.2 Cannibalism

Eight samples (8.0%, *n* = 101) from the forest‐steppe study area and five samples (7.2%, *n* = 69) from the subalpine study area (Table 2) presented lynx remnants, including hair, bones, and claws, suggesting consumption of lynx carcasses rather than just documenting self‐grooming (*n* = 3 removed). In the forest‐steppe study area, one sample was collected in autumn 2013 and seven in spring 2014, and the samples in the subalpine study area were collected in spring and autumn of 2010–2014. No such evidence was found in the Mediterranean study area. This is a conservative assessment of the contribution of cannibalism to the diet as we considered that the feces with lynx hair making <50% of contents could be attributed to self‐grooming.

### 3.3 Prey preferences

In all three study areas, brown hare was the single dominant preferred prey among several other prey species (Table 3). Even in the presence of high available biomass of ungulates, as in the forest‐steppe study area with red deer (24.9% of prey biomass) and in the subalpine study area with wild goat (74.7% of prey biomass), Chesson's α for lagomorphs was 0.90 and 0.99, respectively. In the forest‐steppe study area, red deer was avoided even though it constituted the second highest available biomass among the three most common prey species (Table 3). Moreover, in the subalpine study area, brown hare biomass was available at only 4.22 kg/km^2^ (4.69%), but comprised 77.6% of the diet of lynx in this study area. Hence, Chesson's resource selection index showed strong avoidance of wild goat and red deer where they occurred. Wild boar was avoided as prey species in all three study areas (Table 3).

**Table 3:** Herbivore prey biomass and selectivity in lynx diet in Turkey

	Prey species	Mediterranean	Forest‐steppe	Subalpine
Biomass in diet (%)	Brown hare	**98.52**	**84.76**	**77.58**
	Wild goat	0.0	n.p.	8.2
	Wild boar	1.04	2.84	1.48
	Red deer	n.p.	0.0	n.p.
Biomass available (kg/km^2^)	Brown hare	**114.70**	**280.08**	4.22
	Wild goat	2.10	n.p.	**67.20**
	Wild boar	24.60	80.40	18.60
	Red deer	n.p.	119.25	n.p.
Biomass available (%)	Brown hare	**81.12**	**58.38**	4.69
	Wild goat	1.49	n.p.	**74.65**
	Wild boar	17.40	16.76	20.66
	Red deer	n.p.	24.86	n.p.
Chesson's α	Brown hare	**0.95**	**0.90**	**0.99**
	Wild goat	0.00	n.a.	0.01
	Wild boar	0.05	0.10	0.004
	Red deer	n.a.	0.00	n.a.

*Note*

n.p., not present; n.a., not applicable; highest values are shown in bold.

### 3.4 Functional response of Eurasian lynx, Canada lynx, and Iberian lynx populations to prey

**Figure 4:**
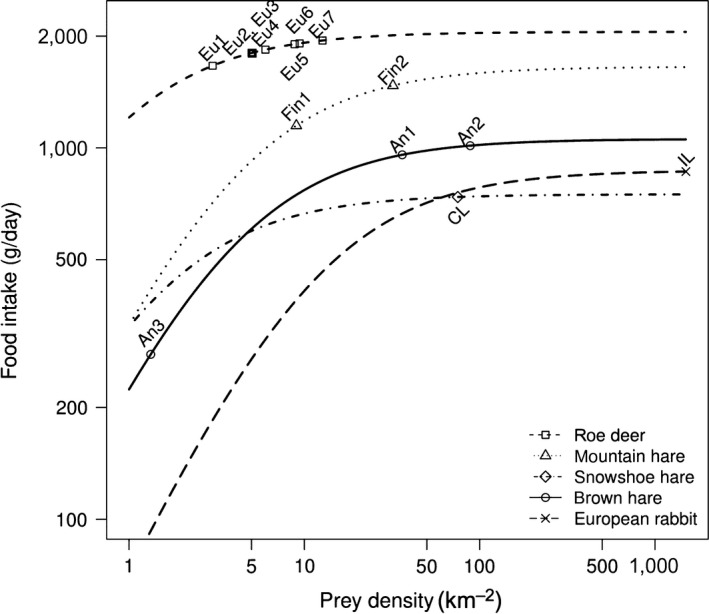
Functional response of Eurasian lynx in Turkey (An1‐An3), Eurasian lynx in central and eastern Europe (Eu1‐Eu7) and in Finland (Fin1, Fin2), Canada lynx (CL), and Iberian lynx (IL) to their prey

Ψ was not highly sensitive to any parameter. The most influential parameter was digestion time (parameter *h*_*2*_), with the 10% change in input indicating a 10% change in output (Ψ). Lynx in Turkey, Canada lynx, and Iberian lynx exhibited a largely similar functional response pattern (similar to type II) and reached close values of asymptotic food intake (900–1,000, 700–800, and 800–900 g/day, respectively) at high prey densities, whereas Eurasian lynx populations in Europe showed a different pattern (Figure 4). Here, asymptotic prey intake was already reached at relatively low roe deer densities (at three individuals per km^2^), substantially earlier than in other functional responses. Food intake of Finnish populations of Eurasian lynx consuming mountain hare (1,400–1,500 g/day) was above these three, but below the seven Eurasian lynx populations which preferentially consume roe deer in central and eastern Europe, where asymptotic prey intake was at its highest level (1,900–2,000 g/day).

## 4 DISCUSSION

The results of this study allowed us to document and understand the importance of high variability in feeding adaptations of a widespread felid species, the Eurasian lynx. Considering the entire distribution of this species, populations in Turkey are geographically closer to European than to Asian populations, yet they show very different dietary patterns.

Mammals are the most important prey category in the diet of lynx in three study areas in Turkey, comprising more than 90% of the diet in average. This result was in accordance with other Eurasian lynx populations throughout its distribution range except for east Siberian populations where birds also contributed significantly (Sedalischev et al., 2014). In contrast to most Eurasian lynx populations in Europe, lynx populations in Turkey strictly relied on brown hare, which formed in average 87% of prey biomass in their diet, even in the presence of mid‐sized or large herbivores such as wild goat, chamois, red deer, and wild boar. In forest‐steppe and subalpine study areas, high biomass of wild goat and red deer did not affect dietary preferences of lynx in Turkey. In these areas, wild goat contributed only 8.2% of prey biomass and red deer was absent in the diet, and thus, both species seemed to be avoided. The only ungulate species which contributed to the diet in all three study areas was wild boar, but it did not exceed 3% in any area. Together with cannibalised lynx, other carnivore species were the second most important food category in the diet of lynx in Turkey. Livestock (domestic goats) were consumed by lynx only in the subalpine area in amounts of 4.4% of prey biomass. However, our data do not allow us to determine whether this contribution resulted from depredation or scavenging.

A similar focus on lagomorph prey preferences was described in six lynx populations in Yakutia, Siberia, with mountain hare making 70% of FO in lynx feces in three areas where hare densities were high (Sedalischev et al., 2014). Sedalischev et al. (2014) suggested that in the areas where mountain hare densities were low, musk and roe deer, young of red deer, moose, and reindeer together with birds contributed more to the diet (20% and 25% of frequencies of occurrences for total deer and birds, respectively). Also, in two other populations in northern Asia and northwest Russia, lagomorphs substantially contributed to the diet with more than 35% of FO (Sedalischev et al., 2014). In none of those three study areas did wild ungulates occur in more than 10% of fecal samples, with the exception of semidomestic reindeer **(** *Rangifer tarandus*) which occurred at 17% in the northern Asia study area (Sedalischev et al., 2014). Similarly, diet of lynx populations in Tibet (53% FO) and north China (81% FO) is mainly composed of lagomorphs (Weidong, 2010), although in Tibet, lynx lived in sympatry with Tibetan gazelles (*Procapra picticaudata*), Tibetan antelope (*Pantholops hodgsonii*), and blue sheep (*Pseudois nayaur*), and in China, lynx lived in sympatry with red deer and roe deer. Ungulates only formed 20% and 11% of frequencies of occurrence in Tibet and north China, respectively (Weidong, 2010).

### 4.1 Foraging preferences and phylogeography of Eurasian lynx

Anatolia was a refuge for many species during the last glacial periods, including brown hare and the Eurasian lynx (Rueness, Naidenko, Trosvik, & Stenseth, 2014; Stamatis et al., 2008). Cold and dry climatic conditions supported the expansion of steppes rather than forests (Atalay, 1998) and, in turn, encouraged the range expansion of brown hare which is still present in most Turkish habitats except for the northern deciduous forests. Other steppe dwelling animals such as Anatolian souslik (*Spermophilus xanthoprymnus*) have also expanded their ranges during these periods (Gür, 2013). We therefore suggest that the high preference of lynx in Turkey for lagomorph prey rather than mid‐sized ungulates regardless of their densities and distributions, and its presence in drier habitats but not in humid deciduous forests (Soyumert, 2010), was a result of a joint biogeographical history which may have resulted in an evolutionary adaptation in terms of foraging specialisation.

Lynx populations in Europe have a decreasing trend of available lagomorph biomass in diet from north to south, most probably due to differential habitat preference patterns of lagomorph species occupying these habitats (Jedrzejewski et al., 1993). In contrast to the co‐occurrence of mountain hares and lynx in boreal forests of northeastern Europe, in central and southern Europe, brown hare occurs mostly in farming areas, open habitats, and forests with many openings and thus are absent in most lynx habitats (Jedrzejewski et al., 1993). The restriction of Eurasian lynx to densely forested habitats in central and southern Europe may have been a consequence of anthropogenic influence, which forced lynx populations out of more open habitats and made the lynx a “refuge species” of forests here as in the case of European bison (*Bison bosanus*) (Kerley, Kowalczyk, & Cromsigt, 2011). Jedrzejewski et al. (1993) pointed out that density of hare and its contribution to lynx diet was higher where there were more forest openings than when there was dense pristine deciduous forest. On the other hand, in the same study, they suggested that lagomorph contribution to lynx diet decreases from northern to southern latitudes. This might be true for Europe, but does not apply to lynx populations elsewhere. Our study and other studies elsewhere in Asia (e.g., Sedalischev et al., 2014; Weidong, 2010) showed that further south and east, in Turkey, Tibet, northeast China, and Siberia, lynx diet was mainly composed of lagomorphs. Therefore, it is likely that the very low contribution of brown hare to lynx diet in central and southern Europe is a consequence of different habitat use by these two species forced by anthropogenic pressures. High densities of forest ungulates and very low densities of lagomorphs in central and southern Europe might be the main cause of dietary specialisation of local lynx populations on ungulates.

### 4.2 Cannibalism and intraguild predation

Most of the fecal samples which consisted of lynx remains in the forest‐steppe study area were collected during the mating and spring (March–May) seasons and in the subalpine study area in spring and early autumn. This time period is crucial for survival of juvenile lynx, as it is when they separate from their mother and begin their own solitary life looking for a new place to live (Schmidt, 1998) and when adult male lynx become aggressively defensive of their territory during mating season (Mattisson et al., 2013). In a high‐density lynx population where many floaters meet many territorial individuals, the chance of encountering a superior conspecific and hence of death is higher (Avgan et al., 2014). Death can take place due to direct killing or injuries resulting from aggressive encounters. Although intraspecific killing can take place in Eurasian lynx behavior (Andrèn et al., 2006; Mattisson et al., 2013), only two cannibalism events in the wild were previously recorded in Eurasian lynx, in Finland (Pulliainen et al., 1995) and the Kostroma region in Russia (Zaitsev, 2009).

Our data suggest that intraspecific killing and cannibalism might be a regular occurrence in lynx populations in Turkey for several reasons: First, we encountered this behavior in two different ecosystems independently (*n* = 13). Second, the lynx feces that included lynx remains in forest‐steppe study area (*n* = 8) were coming from three male and four female individual territories (GPS tracking, Mengüllüoglu, D. unpublished data). Third, six of these samples were genetically (12 microsatellites) identified to originate from five different male and one female individuals. And finally, we encountered high numbers of lynx (27 individuals identified with the help of 12 microsatellites, Mengüllüoğlu, D. unpublished data) in an area of 400 km^2^ during a period of 3 months when those samples were collected. Therefore, the evidence we obtained does not suggest this to be a rare behavior from very few individuals. It may be that cannibalism here was likely to originate from a high lynx density and probable resource and space competition. However, we are not sure whether cannibalism was a result of killing conspecifics for the purpose of feeding or killers made the best out of a bad situation.

Interspecific consumption of other carnivore species by lynx was also recorded in the diet of the central‐western and subalpine study areas, which included golden jackal, domestic dog, and domestic cat in the forest‐steppe study area and red fox and stone marten (*Martes foina*) in the subalpine study area. This is the first report of Eurasian lynx consuming a golden jackal. Our method of dietary analysis using fecal samples does not allow us to distinguish whether these carnivores were scavenged or depredated. However, the lynx is unlikely to be limited to scavenging golden jackals or red foxes, because it is known to kill and eat red foxes (Odden et al., 2006 **),** racoon dogs, and domestic dogs (Okarma et al., 1997) as mesopredator prey or kill and leave the dead bodies (Jobin et al., 2000). If it is correct to assume that Eurasian lynx not only kill red foxes but also golden jackals, then there is the possibility that they may influence the population dynamics of more than one mesopredator. In case of the red fox, Eurasian lynx has the capacity to influence its population dynamics (Sunde, Overskaug, & Kvam, 1999). It is at least conceivable that this may also apply to golden jackals where they are sympatric. Perhaps the recent expansion of golden jackals from southeastern Europe into central Europe might have been encouraged not only by the absence (or reduced presence) of gray wolves (Krofel, Giannatos, Ćirović, Stoyanov, & Newsome, 2017) but also the absence of Eurasian lynx across many central European ecosystems and the restriction of Eurasian lynx to forested habitats in this region.

### 4.3 Prey preferences

Our data from three different lynx habitats, where lynx is in sympatry with at least two ungulate species, showed that even when the biomass of brown hare was lower than the biomass of mid‐sized and large ungulate species, lynx selectively preyed on brown hare (Table 3). This contrasts with the foraging ecology of Eurasian lynx in central and eastern Europe where even juvenile lynx (~12 kg body size) prey on fully grown medium‐sized ungulates, such as roe deer and also on fawns, yearlings, and females of red deer (Okarma et al., 1997). Red deer was totally avoided in the forest‐steppe study area where neither adult and juvenile deer nor calves were consumed by the lynx population. Wild boar was part of the diet in all three study areas, but was clearly avoided in relation to its abundance as demonstrated by low values of Chesson's α (Table 3). The wild boar remains in the analyzed samples were probably scavenged after the “drive hunts” by local hunters for population control, when carcasses are generally left untouched, as the meat is not eaten due to religious beliefs. In two cases, Eurasian lynx was reported to feed on wild boar carrions killed by hunters in winter time (Radikal 2012). Wild goat was the only ungulate species which contributed more than 5% of consumed biomass in lynx diet in the subalpine study area. However, considering the available biomass of this species as a prey source, its percentage in the diet did not indicate any preference by lynx.

### 4.4 Functional response

As suggested by the type II functional response curve, lynx in Turkey had approximately half of the asymptotic prey intake rate of European lynx populations which feed on roe deer (~950 and ~1,800 g/day, respectively). This lower intake rate is in concordance with the smaller body size of lynx in Turkey. The only lynx population which had a very low main prey intake (220 g/day) was the subalpine lynx population (An3). The low prey intake of lynx in subalpine area might originate either from low capture rates, or from low density of hares. We think that the low capture rate of brown hare in subalpine study area was most likely a result of very slow trigger speed (4 s) of the camera trap model used in that study. In case of a really low population density of hares, lynx diet in the subalpine ecosystem would hardly be composed of 78% brown hare in biomass and lynx here would shift to alternative prey sources with higher available biomass (such as wild goat, Table 3), unless this predator population is strictly a lagomorph specialist. Indeed, subalpine lynx population had a higher share of ungulate, bird, and rodents in their diet than the two other lynx populations in Turkey, but still selectively preyed on brown hare like a typical lagomorph specialist (Elton & Nicholson, 1942; Stenseth, Falck, Bjornstad, & Krebs, 1997).

As reported by previous studies on Eurasian lynx populations in central and eastern Europe, the asymptotic intake level was reached quickly even at low roe deer densities and lynx consumed around 1,800 g (mean = 1,836 ± 94 g) of meat per day (Nilsen, Linnell, Odden, & Andersen, 2009; Okarma et al., 1997). Eurasian lynx populations in central and eastern Europe have larger home range sizes (Herfindal, Linnell, Odden, Birkeland Nilsen, & Andersen, 2005) than lynx populations in Turkey (Avgan et al., 2014; Mengüllüoğlu, D. unpublished data), consistent with the idea that there is a negative correlation between the size of a home range and the density of the major prey (Herfindal et al., 2005). Although the search time might increase at lower prey densities, this seemed to matter little as roe deer killing rates in different populations were similar (5–6 days per roe deer), resulting in little differences in food intake rates (Figure 4, Supporting information Table A2).

As shown by the similarity of the type II functional response curves of lynx in Turkey, Canada lynx, and Iberian lynx, we suggest that lynx in Turkey has specialized on a lagomorph diet. This foraging preference may be facilitated by adaptations to hunting brown hares, such as a smaller lynx body size of 9–16 kg in Turkey (Mengüllüoglu, D. unpublished data), and higher population densities at 4.2 individuals/100 km^2^ (Avgan et al., 2014) than elsewhere in Europe where densities are more like 0.4 individuals/100 km^2^ in Germany (Weingarth, Knauer, Scharf, Zimmermann, & Heurich, 2012) or 3.4 individuals/100 km^2^ in Poland (Okarma et al., 1997). If this is the case, we would expect the present distribution of lynx in Turkey to show a considerable overlap with that of brown hare, and a little overlap between lynx and roe deer. Pine forests, forest‐steppe ecosystems, and alpine regions in Turkey appear to provide good habitats to maintain brown hare populations at high densities. These are the areas where lynx are present and live in sympatry with the brown hare in Turkey (Ambarlı, Mengulluoglu, & Bilgin, 2010; Avgan et al., 2014; Capitani, Chynoweth, Kusak, Çoban, & Şekercioğlu, 2016). On the other hand, despite thousands of trap‐days of camera trapping (Özkazanç, Horasan, & Ateşoğlu, 2017; Soyumert, 2010) not a single photograph of a lynx has ever been registered in temperate deciduous ecosystems where roe deer is present at high densities and brown hare is very rare, such as the central Black Sea forests 100 km to forest‐steppe study area. Such a match in predator–prey distributions, specialized diet and prey preferences in three major ecosystems of Turkey suggest that in Turkey the lynx is a lagomorph specialist felid.

### 4.5 Peculiarities of a specialist diet

Eurasian lynx populations in central and eastern Europe are adapted to their main prey, roe deer, by having larger body size and low population density in comparison with lagomorph specialist populations of this species. Therefore, these populations may have a lower chance of encountering prey, have an increased search time and radius, may have to defend larger territories, face a potentially dangerous opponent prior to a successful kill, and then may have to defend kills from kleptoparasitism by other carnivores. Kleptoparasitism is a common phenomenon in Eurasian lynx populations in central and eastern Europe where lynx kills are regularly scavenged or stolen by other predators such as red foxes, martens, brown bears, and even people (Haglund, 1966; Krofel, Kos, & Jerina, 2012). Given all this time and effort, efficacy of food acquisition is also reduced as lynx consumes only flesh making up to 70% of carcass mass (Okarma et al., 1997; Rühe et al., 2007; Sunde et al., 2000).

Previous studies have shown that lagomorph specialist lynx species can experience high population fluctuations following fluctuations in prey densities (Canada lynx: Elton & Nicholson, 1942; Stenseth et al., 1997) or reach the brink of extinction due to prey shortage (Iberian lynx: Ferrer & Negro, 2004). Yet, lagomorph populations can reach very high densities and then reward specialist predators with a rich supply of food. Also, kleptoparasitism is irrelevant as lagomorph specialist lynx can immediately take their kill away from a kill site and consume it within a short period of time. Lynx carrying killed hares in a mouth are not uncommon on camera trap photographs and personal observations in Turkey (Figure 2).

### 4.6 Conservation implications

Our results demonstrated that the diet of the Eurasian lynx in Turkey consists mostly of brown hares and that its foraging ecology fulfills expectations for a lagomorph specialist, similar to Iberian and Canada lynx, regardless of ecosystem. This result is in sharp contrast to what would be expected from the generalization of feeding ecology of lynx in Europe over larger scales. Our studies are also consistent with previous results of Asian populations of the Eurasian lynx, which also strongly rely on lagomorphs (Sedalischev et al., 2014; Weidong, 2010).

To become efficient, lynx conservation programmes in southwest Asia should be implemented in areas with moderate‐to‐high densities of lagomorphs and clearly address the status, threats, and factors related to these species. Any rewilding projects undertaken in southwest Asia should consider using individuals from lynx populations from Turkey rather than from Europe where lynx rely on ungulate prey. First, this will ensure that the lynx will adequately cope with the local prey base. Second, this will increase public acceptance and minimize the potential for conflict with farmers because predation on domestic livestock by lynx in Turkey is very rare in contrast to predation on domestic livestock by lynx in central and eastern Europe (Odden et al., 2006).

## CONFLICT OF INTEREST

None declared.

## DATA ARCHIVING

The data were given in Tables, and the R scripts for Holling's disc equation were given as a Supporting information Appendix S1.

## AUTHOR CONTRIBUTIONS

DM, HA, AB, and HH designed the study. HA and DM conducted field works. DM and HA performed laboratory analysis and calculations. DM and HA wrote the drafted manuscript. HH and AB provided input to the writing of the manuscript. HA, DM, HH, and AB revised the final manuscript. HA is the corresponding author.
