# Supplementary material for: Foraging ecology of Eurasian lynx populations in southwest Asia: Conservation implications for a diet specialist
Source: Ecol Evol. 2018 Aug 29;8(18):9451–63. doi: 10.1002/ece3.4439 (PMC6194280; doi:10.1002/ece3.4439)
Supplement: Supplementary file 1 [file ECE3-8-9451-s001.docx]

**Supplementary Material**

**Table A1.** Reference hair collection used in prey species identification in diet analyses (√ available to us, x not available to us).

|  | **Latin name** | **Species name** | **Adult** | **Young** | **Winter coat** | **Summer coat** | **Source** |
| --- | --- | --- | --- | --- | --- | --- | --- |
| Erinaceidae | *Erinaceus concolor* | Southern white-breasted hedgehog | **√** | x | x | x | Berlin Natural History Museum |
| Sciuridae | *Sciurus anomalus* | Caucasian squirrel | **√** | x | x | x | Berlin Natural History Museum |
|  | *Sciurus vulgaris* | Red squirrel | **√** | x | x | x | Berlin Natural History Museum |
| Gliridae | *Dryomys nitedula* | Forest dormouse | **√** | x | x | x | Berlin Natural History Museum |
| Cricetidae | *Microtus guentheri* | Guenther's vole | **√** | x | x | x | Berlin Natural History Museum |
| Muridae | *Meriones tristrami* | Tristram's jird | **√** | x | x | x | Berlin Natural History Museum |
|  | *Apodemus sylvaticus* | Wood mouse | **√** | x | x | x | Berlin Natural History Museum |
|  | *Mus musculus* | House mouse | **√** | x | x | x | Berlin Natural History Museum |
|  | *Rattus rattus* | Black Rat | **√** | x | x | x | Berlin Natural History Museum |
| Leporidae | *Lepus europaeus* | Brown hare | **√** | x | x | x | Berlin Natural History Museum |
| Felidae | *Felis chaus* | Jungle cat | **√** | x | x | x | Berlin Natural History Museum |
|  | *Felis silvestris* | Wild cat | **√** | x | x | x | Berlin Natural History Museum |
|  | *Lynx lynx* | Eurasian lynx | **√** | x | x | x | Berlin Natural History Museum |
| Canidae | *Vulpes vulpes* | Red fox | **√** | x | x | x | Berlin Natural History Museum |
|  | **Latin name** | **Species name** | **Adult** | **Young** | **Winter coat** | **Summer coat** | **Source** |
|  | *Canis aureus* | Golden jackal | **√** | x | x | x | Berlin Natural History Museum |
|  | *Canis lupus* | Wolf | **√** | x | x | x | Berlin Natural History Museum |
| Mustelidae | *Martes foina* | Stone marten | **√** | x | x | x | Berlin Natural History Museum |
|  | *Mustela nivalis* | Least weasel | **√** | x | x | x | Berlin Natural History Museum |
|  | *Vormela peregusna* | Marbled polecat | **√** | x | x | x | Berlin Natural History Museum |
|  | *Meles meles* | Eurasian badger | **√** | x | x | x | Berlin Natural History Museum |
| Suidae | *Sus scrofa* | Wild boar | **√** | **√** | **√** | **√** | Berlin Natural History Museum |
| Cervidae | *Capreolus capreolus* | Roe deer | **√** | **√** | **√** | **√** | Berlin Natural History Museum |
|  | *Cervus elaphus* | Red deer | **√** | **√** | **√** | **√** | Berlin Natural History Museum |
| Bovidae | *Ovis gmelinii anatolica* | Anatolian wild sheep | **√** | **√** | **√** | **√** | Study area |
|  | *Capra aegagrus* | Wild goat | **√** | **√** | **√** | **√** | Study area |
|  | *Ovis musimon* | Domestic sheep | **√** | **√** | **√** | **√** | Study area |
|  | *Capra hircus* | Domestic goat | **√** | **√** | **√** | **√** | Study area |

**Table A2.** Parameters used in Holling’s Disc Equation and daily food intake rates calculated for twelve Eurasian, one Canada and one Iberian lynx populations.

| **ID** | **Population** | **Main prey** | **Average adult prey mass (g)** | **Prey density (km2)** | **Path width (km)** | **Lynx travel speed (km/h)** | **Success of attack** | **Time per attack (sc)** | **Digestive time (days)** | **Calculated prey intake (g/day)** |
| --- | --- | --- | --- | --- | --- | --- | --- | --- | --- | --- |
| An1 | Mediterranean-TR | brown hare | 3170**^1^** | 37.5**^6^** | 0.05 | 0.213**^7^** | 0.35**^1^**^7^ | 33**^17^** | 3**^7^** | 958 |
| An2 | Forest-Steppe-TR | brown hare | 3170**^1^** | 88.8**^7^** | 0.05 | 0.213**^7^** | 0.35**^17^** | 33^17^ | 3**^7^** | 1014 |
| An3 | Subalpine-TR | brown hare | 3170**^1^** | 1**^8^** | 0.05 | 0.213**^7^** | 0.35**^17^** | 33^17^ | 3**^7^** | 278 |
| Eu1 | Norway* | roe deer | 17600**^2^** | 3**^9^** | 0.05 | 0.3**^16^** | 0.66**^17^** | 33^17^ | 6 | 1664 |
| Eu2 | Norway** | roe deer | 17600**^2^** | 5**^9^** | 0.05 | 0.3**^16^** | 0.66**^17^** | 33^17^ | 6 | 1800 |
| Eu3 | Bohemian | roe deer | 17600**^2^** | 5**^10^** | 0.05 | 0.3**^16^** | 0.66**^17^** | 33^17^ | 6 | 1800 |
| Eu4 | Dinaric | roe deer | 17600**^2^** | 6**^11^** | 0.05 | 0.3**^16^** | 0.66**^17^** | 33^17^ | 6 | 1838 |
| Eu5 | Jura | roe deer | 17600**^2^** | 8.8**^12^** | 0.05 | 0.3**^16^** | 0.66**^17^** | 33^17^ | 6 | 1901 |
| Eu6 | Bialowieza | roe deer | 17600**^2^** | 9.4**^13^** | 0.05 | 0.3**^16^** | 0.66**^17^** | 33^17^ | 6 | 1910 |
|  |  |  |  |  |  |  |  |  |  |  |
|  |  |  |  |  |  |  |  |  |  |  |
| **ID** | **Population** | **Main prey** | **Average adult prey mass (g)** | **Prey density (km2)** | **Path width (km)** | **Lynx travel speed (km/h)** | **Success of attack** | **Time per attack (sc)** | **Digestive time (days)** | **Calculated prey intake (g/day)** |
| Eu7 | Swiss Alps | roe deer | 17600^2^ | 12.7**^14^** | 0.05 | 0.3**^16^** | 0.66**^17^** | 33**^17^** | 6 | 1945 |
| Fin1 | Finland East | mountain hare | 3308**^3^** | 9**^15^** | 0.05 | 0.3**^16^** | 0.35**^17^** | 33**^17^** | 2 | 1147 |
| Fin2 | Finland south-east | mountain hare | 3308**^3^** | 32**^15^** | 0.05 | 0.3**^16^** | 0.35**^17^** | 33**^17^** | 2 | 1471 |
| Cl | Yukon  Canada lynx | snowshoe hare | 1500**^4^** | 75**^4^** | 0.05 | 1.09**^4^** | 0.30**^4^** | 33**^17^** | 2 | 737 |
| Il | Donana  Iberian lynx | European rabbit | 870**^5^** | 1500**^5^** | 0.05 | 0.3**^5^** | 0.25**^5^** | 30**^4^** | 1 | 863 |
|  |  |  |  |  |  |  |  |  |  |  |

**Norway medium roe deer density

* Norway low roe deer density

**References**

**^1^**Demirbaş, Y. et al. 2013. Studies of ecomorphological variations of the European hare (Lepus europaeus) in Turkey. Arch. Biol. Sci. Belgrade 65:559-566

**^2^**Mysterud and Ostbye 2006. Effect of climate and density on individual and population growth of roe deer Capreolus capreolus at northern latitudes: the Lier valley, Norway. Wildl. Biol., 12(3):321-329

**^3^**Kauhala et al. 2005. Population dynamics of mountain hare Lepus timidus populations in Finland. - Wildl. Biol. 11: 299-307

**^4^**O'Donoghue M. et al. 1998. Responses of Coyotes and Lynx to the Snowshoe Hare Cycle. Ecology, 79(4): 1193-1208

**^5^**Aldama J.J. et al. 1991. Energy Expenditure and Prey Requirements of Free-Ranging Iberian Lynx in Southwestern Spain. J. Wildl. Man., 55(4): 635-641

**^6^**Avgan B. et al. 2014. The first density estimation of an isolated Eurasian lynx population in southwest Asia. Wildl. Biol. 20: 217-221

**^7^**Mengüllüoğlu, unpublished data.

**^8^**Ambarlı H. and Bilgin C.C. 2013. First record of a melanistic golden jackal (Canis aureus, Canidae) from Turkey. Mammalia, 77: 219-222

**^9^**Linnell J.D.C. et al. (2007). Distance rules for minimum counts of Eurasian lynx Lynx lynx family groups under different ecological conditions. Wildl. Biol. 13(4):447-455

**^10^**Heurich M. et al. (2012). Survival and causes of death of European Roe Deer before and after Eurasian Lynx reintroduction in the Bavarian Forest National Park. Eur. J. Wildl. Res., 58(3): 567–578

**^11^**Krofel M. et al. (2014). Comparing patterns of human harvest and predation by Eurasian lynx Lynx lynx on European roe deer Capreolus capreolus in a temperate forest. Eur. J. Wildl. Res. 60(1): 11-21

**^12^**Jobin, A. et al. (2000). Prey spectrum, prey preference and consumption rates of Eurasian lynx in the Swiss Jura Mountains. Acta Theriol. 45: 243-252

**^13^**Jędrzejewski W. et al. 1993. Foraging by lynx and its role in ungulate mortality: the local (Białowieża Forest) and the Palaearctic viewpoints. Acta Theriol. 38: 385-403.

**^14^**Breitenmoser U. and Haller H. 1993. Patterns of Predation by Reintroduced European Lynx in the Swiss Alps. J. Wildl. Man., 57(1): 135-144

**^15^**Kauhala K. et al. 1999. Impact of predator removal on predator and mountain hare populations in Finland. Annl. Zool. Fennici, 36(3): 139-148

**^16^**Jędrzejewski W. et al. 2002. Movement pattern and home range use by the Eurasian lynx in Bialowieza Primeval Forest (Poland). Ann. Zool. Fennici, 39: 29–41

**^17^**Pulliainen E. 1981. Winter diet of Felis lynx L. in SW Finland as compared with the nutrition of other northern lynxes. Z. Sáugetierk 46: 249-259
